# Clinical Significance of Detection of Coagulation Indexes, Immune Factors and Inflammatory Factors in Patients with Pregnancy-Induced Hypertension Syndrome in China

**Published:** 2019-04

**Authors:** Fengmei SHI, Aijun YU, Limei YUAN

**Affiliations:** 1. Clinical Laboratory of Weihaiwei People's Hospital, Weihai, Shandong Province, China; 2. Department of Obstetrics and Gynecology, Dezhou People’s Hospital, Dezhou, China; 3. Department of Gynecology and Obstetrics, The Fifth People’s Hospital of Jinan, Jinan, China

**Keywords:** Coagulation index, Immune factor, Pregnancy-induced hypertension syndrome

## Abstract

**Background:**

To investigate the clinical significance of monitoring the coagulation indexes, immune factors and inflammatory factors in pregnancy-induced hypertension syndrome (PIH).

**Methods:**

90 pregnant women with PIH admitted in Weihaiwei People's Hospital of Shandong Province, China from 2016 to 2017 were collected, including 45 cases in mild-moderate group and 45 cases in severe group. Another 45 normal pregnant women at the same period were selected as control group. The immune indexes, four index signs of coagulation bloods and serum inflammatory factors in three groups of subjects were determined.

**Results:**

The levels of complement 3 (C3), complement 4 (C4), immunoglobulin A (IgA), immunoglobulin G (IgG) and immunoglobulin M (IgM) were successively increased in severe PIH group, mild-moderate PIH group and normal pregnancy group (*P*<0.05). Compared with those in normal pregnancy group, the levels of PT, APTT and TT were significantly decreased and Fib levels were significantly increased in PIH groups (*P*<0.05). Compared with those in normal pregnant women, the levels of TNF-α and IL-6 in pregnant women with PIH were significantly increased (*P*<0.05), and the changes in severe PIH group were more obvious than those in mild-moderate group.

**Conclusion:**

Pregnant women with PIH are in a hypercoagulable state and have a higher risk of thrombus and secondary hyperfibrinolysis. Immune factors and inflammatory factors are also associated with the occurrence and development of the disease. Monitoring the changes in coagulation indexes and levels of immune factors and inflammatory factors provide an important reference value for clinical treatment and prevention of complications.

## Introduction

Pregnancy-induced hypertension syndrome (PIH) is a kind of high risk pregnancy with hypertension, proteinuria and edema as the main clinical manifestations, and systemic small vasospasm and vascular endothelial injury as the pathological features. It can eventually cause multiple organ failure due to coagulation/fibrinolysis system imbalance (1–3). The pathogenesis mainly includes placental pregnancy hypertension theory, immunological theory, placental cytokine theory and maternal pregnancy hypertension theory (4). The purpose of this study was to investigate the clinical characteristics of the occurrence and development of PIH from the point of view of coagulation indexes, immune factors and inflammatory factors, in order to understand the relationships of them with the condition of PIH, so as to provide a practical basis for clinical diagnosis, prevention and treatment of PIH.

## Methods

### Research objects and grouping

A total of 90 pregnant women with PIH admitted in Weihaiwei People's Hospital of Shandong Province (Weihai, China) from January 2016 to January 2017 were collected, including 45 cases in mild-moderate group and 45 cases in severe group. Diagnostic criteria were based on those in Obstetrics and Gynecology (5). Patients with chronic hypertension, diabetes, heart disease, primary renal disease or other pregnancy complications were excluded. Patients were randomly divided into normal late pregnancy group, mild-moderate PIH group and severe PIH group, with 45 cases in each group. The general data [age, gender, body mass index (BMI), etc.] in three groups were comparable.

The study was approved by the Ethics Committee of Weihaiwei People's Hospital of Shandong Province, China. Patients and their families signed the informed consent before the start of the study.

### Research methods

A total of 5 mL fasting elbow venous blood was extracted from the subjects in early morning, and 2 mL was taken out and placed in a vacuum tube for rapid serum separation for the detection of immune indicators and inflammatory factors. 3 mL blood sample was taken out and added with 0.2 mL sodium citrate (0.109 moL/L), placed in a disposable anticoagulant tube, and centrifuged to separate the plasma for the detection of coagulation indexes.

### Detection of immune indexes

Immunoturbidimetry was used to detect immune indexes of the subjects, including complement 3 (C3), complement 4 (C4), immunoglobulin A (IgA), immunoglobulin G (IgG) and immunoglobulin M (IgM). The 7600 full-automatic biochemical analyzer (Hitachi, Japan) was used for detection.

### Coagulation indexes

The full-automatic coagulation analyzer (Stago, France) was used for the detection of plasma coagulation indexes, including prothrombin time (PT), activated partial thromboplastin time (APTT), thrombin time (TT) and fibrinogen (Fib).

### Detection of inflammatory factors

The 7600 full-automatic biochemical analyzer (Hitachi, Japan) was used for detection.

### Clinical data

The clinical data of patients were collected, and univariate and multivariate analyses on the relationships between various influencing factors and the incidence of PIH were carried out.

### Statistical methods

Statistical Product and Service Solutions (SPSS) 19.0 statistical software (IBM) was used to process data results. Measurement data were expressed as mean ± standard deviation, and enumeration data were expressed as rate (%). Analysis of variance was used for comparisons among groups, and univariate and multivariate analyses of each influencing factor were carried out. *p*<0.05 suggested that the difference was statistically significant.

## Results

### Comparisons of general conditions among three groups

There were no statistically significant differences in age, gestational weeks and BMI among three groups of pregnant women. The difference between systolic pressure and diastolic pressure was statistically significant (*P*=0.012, *P*=0.023) (Table 1).

**Table 1:** Comparisons of general conditions among three groups (n=45)

***Group***	***Age(yr)***	***Gestational weeks***	***BMI (kg/m^2^)***	***Systolic pressure(mmHg)***	***Diastolic pressure (mmHg)***
Normal pregnancy group	26.8±4.0	38.8±1.2	27.0±1.4	117.7±5.8	71.3±7.1
PIH (mild-moderate group)	27.5±3.8	38.0±1.6	28.0±1.3	150.3±10.2*	100.2±9.8*
PIH (severe group)	27.1±3.3	37.7±1.4	28.5±1.4	168.2±13.8*#	117.7±9.2*#

Note: Compared with that in normal pregnancy group,

* *P*<0.05.

Compared with that in mild-moderate group,

# *P*<0.05

### Comparisons of coagulation indexes among three groups

Compared with those in normal pregnancy group, PT, APTT and TT values in PIH groups were significantly decreased, while Fib levels were significantly increased, and the changes of coagulation indexes in severe group were larger than those in mild-moderate PIH group, with statistically significant differences among three groups (*P*=0.008, *P*=0.003, *P*=0.006) (Table 2).

**Table 2:** Comparisons of coagulation indexes among three groups (n=45)

***Group***	***PT (s)***	***APTT (s)***	***Fib (g/L)***	***TT (s)***
Normal pregnancy group	13.01±0.79	31.56±3.58	3.69±0.78	15.88±3.72
PIH (mild-moderate group)	12.11±0.31*	28.30±2.61*	5.02±0.67*	13.78±4.01
PIH (severe group)	10.77±0.51*#	25.02±2.98*#	6.08±0.86*#	12.12±3.05

Note: Compared with that in normal pregnancy group,

* *P*<0.05.

Compared with that in mild-moderate group,

# *P*<0.05

### Comparisons of immune indexes among three groups

Levels of C3, C4, Ig A, IgG and IgM in three groups: Severe PIH group<mild-moderate group<normal pregnancy group. Compared with those in normal pregnancy group, the levels of C3, C4, IgA, IgG and IgM in severe group were significantly decreased, and the differences were statistically significant (*P*<0.05) (Table 3).

**Table 3:** Comparisons of immune indexes among three groups (n=45)

***Group***	***C3(g/L)***	***C4(g/L)***	***IgA(g/L)***	***IgG(g/L)***	***IgM(g/L)***
Normal pregnancy group	0.93±0.09	0.21±0.12	1.87±0.58	13.25±0.80	1.62±0.28
PIH (mild-moderate group)	0.88±0.11	0.19±0.06	1.83±0.78	12.35±0.89	1.58±0.33
PIH (severe group)	0.65±0.28*	0.17±0.05*	1.60±0.27*	9.68±1.12*	1.21±0.40*

Note: Compared with that in normal pregnancy group,

* *P*<0.05

### Comparisons of serum levels of tumor necrosis factor-α (TNF-α) and interleukin-6 (IL-6) among three groups

The determination of TNF-α and IL-6 were successively decreased in severe PIH group, mild-moderate PIH group and normal pregnancy group, the changes in severe PIH group were more obvious than those in mild-moderate group (*P*=0.004, *P*=0.001) (Table 4).

**Table 4:** Comparisons of serum levels of TN F-α and IL-6 among three groups

***Detection index***	***Normal pregnancy group***	***PIH (mild-moderate group)***	***PIH (severe group)***
TNF-α (ng/L)	1.02±0.13	1.28±0.17*	1.66±0.21*#
IL-6 (ng/L)	70.13±28.33	121.36±46.88*	180.24±50.26*#

Note: Compared with that in normal pregnancy group,

* *P*<0.05.

Compared with that in mild-moderate group,

# *P*<0.05

### Analyses of risk factors for the incidence of PIH

Univariate analysis showed that age of onset, BMI, alcohol consumption and smoking were significantly correlated with the incidence of PIH.

Further multivariate analysis showed that the independent factors of PIH included senility, overweight, alcohol consumption and smoking (Tables 5-6).

**Table 5:** Univariate analysis on the incidence of PIH

***Influencing Factor***	***PIH group (n=90)***	***Normal Pregnancy Group (n=45)***	***X^2^ value***	***P***
Age (yr)			9.633	0.005
20–30	18	10		
30–40	30	28		
> 40	42	7		
BMI			38.966	0.000
BMI≤18.5	11	3		
Normal	22	25		
BMI≥25	57	17		
Alcohol Consumption			41.235	0.000
Yes	40	3		
No	50	42		
Smoking			48.963	0.000
Yes	60	6		
No	30	39		

**Table 6:** Analysis of high risk factors for the incidence of PIH

***Variable***	***Regression coefficient***	***Standard error***	***Wald***	***P***
Senility	0.368	0.298	1.801	0.016
Overweight	0.274	0.089	7.863	0.003
Alcohol consumption	0.712	0.220	9.412	0.000
Smoking	0.799	0.343	8.322	0.001

## Discussion

PIH not only damages the organ function of pregnant women, but also seriously endangers the life of the fetus during the development of eclampsia. After normal pregnancy, coagulation substances and anticoagulant substances change greatly, and the blood enters into a certain hypercoagulable state, which is conducive to postpartum hemostasis. However, there are factors that interfere with the balance of coagulation and fibrinolysis system in patients with PIH, leading to a higher risk of thrombosis (6).

Blood coagulation function test is a more routine test item mainly used in the diagnosis of hemorrhagic diseases. PT is an important index reflecting the condition of exogenous coagulation system (coagulation factors V, VII, X, etc.) (7). APTT reflects the operation of endogenous coagulation system (coagulation factors VIII, IX, XI, etc.) (8). Fib is a glycoprotein derived from the liver, with the highest content among plasma coagulation factors. Fib level is closely related to thrombin activity and is an important factor leading to thrombosis. The higher the level of Fib is, the higher the risk of body thrombosis will be (9). TT refers to the time during which plasma is coagulated by a standardized prothrombin in vitro, and TT is prolonged when heparin-like substances and/or fibrin degradation products in plasma are increased (10). The results of this study showed that compared with those in normal pregnant women, PT, APTT and TT in pregnant women with PIH were significantly decreased, while Fib was significantly increased (*P*<0.05), and the changes in severe PIH group were more obvious than those in mild-moderate group. The results showed that pregnant women with PIH are in a hypercoagulable state and have a higher risk of thrombosis and secondary hyperfibrinolysis.

Monitoring the coagulation system and fibrinolysis system can intervene with prethrombotic state, diffuse intravascular coagulation and other diseases in advance, especially in patients with PIH (11). IgG is the only immunoglobulin that can pass through the placenta, and its function is mainly to protect the immune function of the body (12, 13). PIH is closely related to complement activation, and complement active products activate neutrophils and macrophages to release histamine, proteolytic enzymes, interleukins and other vasoactive substances. In addition, excessive immune complex deposition is also found on the blood vessel wall and trophoblast basement membrane of the liver, kidney and placenta in patients with PIH, and the more serious the disease is, the higher the complex level will be, so the lower the level of antibodies detected in serum will be (14–16). This study showed that serum complement and immunoglobulin levels in patients with PIH were significantly decreased compared with those in normal pregnancy group, indicating that the immune function of patients with PIH is obviously inhibited.

At present, most studies have suggested that fetal-maternal immune imbalance and reduced uterine placenta blood supply induce the release of related cytokines into the blood and produce vasoactive substances, leading to the occurrence of PIH (17). The serum TNF-α level in patients with PIH was significantly higher than that in normal pregnancy group (18), and found that TNF-α is involved in the pathogenesis of eclampsia, which is of great significance for the diagnosis and evaluation of PIH and of great value for the prediction of the incidence of eclampsia. When PIH occurs, the placenta blood perfusion is insufficient and the fetus is ischemic and anoxic, thus inducing TNF-α, IL-6 and other inflammatory factors (19). TNF-α binds to the corresponding receptors on vascular endothelial cells, and activates the oxidative free radicals and their corresponding proteases, causing endothelial cell damage. TNF-α can also induce an increase in the production of endothelin-1 (ET-1) and platelet-derived growth factor (PDGF) in vasoconstrictor factors. IL-6 can increase the concentration of vascular cell adhesion molecule-21 (VCAM-21). These factors constitute the basic pathological basis of PIH (20). It was found in this study that compared with those in normal pregnancy women, TNF-α and IL-6 levels in pregnant women with PIH were significantly increased (*P*<0.05), and the changes in severe PIH group were more obvious than those in mild-moderate PIH group. The results showed that the severity of the disease is related to the levels of inflammatory factors.

The incidence of PIH has been increasing year by year, so scholars pay more attention to its etiology and pathogenesis. At present, the cause and mechanism of the disease are still not fully understood. In summary, there are mainly placental factors and maternal factors. Maternal factors are due to systemic microvascular diseases and susceptibility to certain diseases, which mainly include hypertension, diabetes, obesity and other causes of maternal systemic inflammatory response, leading to disorder of circulatory function (21). The results of this study showed that the age of onset, BMI, alcohol consumption and smoking are significantly correlated with the incidence of PIH. The independent factors of PIH include senility, overweight and obesity, alcohol consumption and smoking.

## Conclusion

Pregnant women with PIH are in a hypercoagulable state and have a higher risk of thrombosis and secondary hyperfibrinolysis. Immune factors and inflammatory factors are also involved in the occurrence and development of the disease. Monitoring the changes in coagulation indexes and levels of immune factors (C3, C4, IgA, IgG and IgM) and inflammatory factors (TNF-α and IL-6) provides an important reference value for the clinical treatment and prevention of complications.

## Ethical considerations

Ethical issues (Including plagiarism, informed consent, misconduct, data fabrication and/or falsification, double publication and/or submission, redundancy, etc.) have been completely observed by the authors.
